# Pediatric Phthiriasis Palpebrarum: A French Multicentric Retrospective Study of 20 Cases

**DOI:** 10.1016/j.jpedcp.2026.200219

**Published:** 2026-05-30

**Authors:** Marie Damez, Anne Welfringer, Christine Leaute-Labreze, Christine Chiaverini, Stephanie Mallet, Mathilde Tardieu, Marie-Helene Jegou, Emmanuel Mahe, Annabel Maruani, Laurent Misery, Claire Abasq-Thomas, Groupe de Recherche de la Société Française de Dermatologie Pédiatrique

**Affiliations:** 1 Department of Dermatology, University Hospital of Brest, Brest, France; 2 Department of Paediatric Dermatology, Assistance Publique Hôpitaux de Paris (APHP), Necker University Hospital, Paris, France; 3 Department of Dermatology, University Hospital Center (CHU) of Bordeaux, Bordeaux, France; 4 Department of Dermatology, University Hospital Center (CHU) of Nice, Nice, France; 5 Department of Dermatology, Assistance Publique-Hôpitaux de Marseille (AP-HM), Marseille, France; 6 Dermatology, Allergology and Photobiology Department – CHU Grenoble Alpes, University Grenoble Alpes, Grenoble, France; 7 Liberal Office of Dermatology, Blanquefort, France; 8 Department of Dermatology, Hôpital Victor Dupouy, Argenteuil, France; 9 INSERM 1246-SPHERE, Unit of Pediatric Dermatology, Department of Dermatology, University Hospital Center (CHRU) of Tours, University of Tours, Tours, France

**Keywords:** child abuse, parasitic, pediatrics, pediculosis ciliare, phthiriasis ciliare, phthiriasis palpebrarum, *Phthirus pubis*, pubic lice

## Abstract

**Objective:**

Phthiriasis palpebrarum is an ectoparasitosis caused by *Phthirus pubis*. We lack data on managing this condition in pediatric populations. The aim of this multicenter retrospective study from France was to describe the clinical characteristics, the modes of transmission, and the treatments of children affected by phthiriasis palpebrarum.

**Study design:**

Records of phthiriasis palpebrarum cases that occurred in children <18 years from 2005 to 2024 were retrieved from 9 dermatology departments and 1 liberal office in France.

**Results:**

A total of 20 children with phthiriasis palpebrarum were included. The median age at diagnosis was 3 years and 2 months (range: 7 months-8 years). The median delay in diagnosis was 28.5 days (range: 1 day-5 months). Ocular pruritus (83%), eyelid erythema (44%), crusting of the free eyelid margin (41%), and scalp involvement (55%) were the main characteristics. A case of *P pubis* infection in close contacts was identified in 12 patients (63%). The mode of contamination was identified in 53% of cases. No case of sexual abuse was found. The majority of patients were treated with ivermectin, topicals, and mechanical removal.

**Conclusions:**

Contrary to the previous reports in the literature about pediatric phthiriasis palpebrarum, scalp involvement is a typical finding. Regarding the mode of transmission, no sexual abuse has been identified, but the origins of infantile phthiriasis palpebrarum weren't systematically discerned to assess for possible abuse. Notably, no case was registered after the age of 8 years. Different treatment protocols are used, reflecting a lack of standardization.

Phthiriasis palpebrarum, also called “phthiriasis ciliaris” or “ciliary phthiriasis,” is an ectoparasitosis of the eyelashes caused by an infestation of “*Phthirus pubis*”, also known as the pubic or crab louse. Infestation usually occurs in adults on the pubis, inguinal folds, buttocks, and perianal region. In patients with a great deal of body hair or when the infestation is longstanding, this louse can also be found on the thighs, abdomen, chest, axillae, and beard.^1^, ^2^, ^3^, ^4^, ^5^

The eyelashes are a classic, although rare, site of *P pubis* infestation in children because of the lack of terminal hair on other parts of the body. The involvement of the scalp by *P pubis* also has been recently reported.^6^^,^^7^ Another species of parasite that is observed with much greater frequency in children is *Pediculus capitis*, an ectoparasite that affects only the scalp. The adult head louse is 2 to 3 mm long and is usually tan to grayish-white in color, whereas *P pubis* has a smaller body of 1.2 × 0.8 mm, may be lighter in color, and is not as mobile.^8^ *P pubis* is transmitted by close contact and is considered a potentially sexually transmitted infection. The origins of this infection in children need to be carefully discerned, and the question of sexual abuse must be raised.^9^^,^^10^ Limited case series exist in the literature, and optimal diagnosis and treatment are lacking.^11^, ^12^, ^13^ The largest pediatric cohort published to date consisted of 5 children, aged between 2 and 5 years. All of the subjects presented with a coinfection of the hair and eyelashes. No cases of sexual abuse were reported in this series.^14^ In this context, the goal of the present study was to delineate the clinical characteristics of phthiriasis palpebrarum in children, investigate the modes of infestation, and collect data on treatments in a retrospective multicenter French cohort.

## Patients and Methods

### Study Setting and Period

A multicenter retrospective cohort was conducted in France that included 9 dermatology hospital departments and 1 private dermatology practice. Case recruitment was carried out through the research group of the French Society for Pediatric Dermatology, and the recruitment period for pediatric phthiriasis palpebrarum cases spanned from May 2023 to April 2024.

### Study Population

Eligibility criteria included patients aged 0-18 years at the time of diagnosis, made by a dermatologist in the past 20 years (between 2005 and 2024), on the basis of clinical findings and/or dermoscopic and/or parasitological identification of *P pubis* on the eyelashes, confirming a diagnosis of phthiriasis palpebrarum.

### Data Collection

Clinical, epidemiologic, and therapeutic data were retrospectively collected by the investigators on the basis of the recorded medical history, using a questionnaire specifically designed for this cohort. Clinical characteristics were collected, including demographic data, presence/absence of pruritus, erythema, crusting on the free margin of the eyelids, or parasitic involvement of the scalp hair associated with ciliary involvement. Diagnostic data included time to diagnosis and supporting diagnostic investigations; treatments were also recorded. The questionnaire was designed to ascertain the method of infection transmission, distinguishing between direct and indirect transmissions via contaminated linen or bedding. Finally, the investigation inquired about the status of sexual abuse allegations: confirmation, suspicion, ruling out, or absence of mention.

### Statistical Analyses

Continuous variables were expressed as medians or means with their minimum and maximum values (range), and categorical variables were expressed as numbers (percentages) . Statistical analyses were performed using Microsoft Excel 2016.

### Ethics Statement

This is a retrospective, noninterventional study. In accordance with the French Public Health Code (art L 1121-1-1, art L 1121-1-2), the approval of an institutional review board was not required. However, all legal representatives of the children included in the study were informed about the use of the child's medical data, in accordance with current French regulations.

## Results

### Demographic Characteristics and Clinical Findings

A total of 20 cases of pediatric phthiriasis palpebrarum were included in the study. Of the 20 included children, 10 were girls and 10 were boys. The median age at diagnosis was 3 years and 2 months (minimum = 7 months, maximum = 8 years). Five patients (25%) were aged <2 years, and 9 (45%) between 2 and 5 years. Fourteen (70%) children had no significant medical history. Of the reported medical history, 2 children (10%) had a history of scalp pediculosis and 1 child (5%) had a history of recurrent conjunctivitis. Other antecedents unrelated to parasitosis or ocular involvement also were reported: syndromic vascular malformation, posthectomy, and unspecified medical history (n = 3%-15%). Characteristics of the patients are shown in the Table.

**Table:** Patient demographics, disease, and treatment characteristics

Characteristics	Number of patients
Total, No. (%)	20 (100)
Age, y, median (range)	3.2 (0.58-8)
<1 y, No. (%)	1 (5)
1 to <2 y, No. (%)	4 (20)
2 to <5 y, No. (%)	9 (45)
≤5 y, No. (%)	6 (30)
Male patients, No. (%)	10 (50)
Delay between symptom onset and diagnosis, d, median (range)	29 (1-152)
Bilateral involvement, No. (%)	16/17 (94)
Scalp involvement, No. (%)	11 (55)
Presence of itch, No. (%)	15/18 (83)
Household members affected, No. (%)	12/19 (63)
Mode of transmission identified, No. (%)	10 (53)
Hospitalization, No. (%)	2 (10)
Treatments	
Topical chemical treatment alone, No. (%)	2 (10)
Ivermectin alone, No. (%)	1 (5)
Mechanical and topical chemical treatment, No. (%)	4 (20)
Mechanical treatment and ivermectin, No. (%)	4 (20)
Topical chemical treatment and ivermectin, No. (%)	1 (1)
Mechanical, topical chemical treatment, and ivermectin, No. (%)	8 (40)
Environmental decontamination, No. (%)	17 (85)

The median delay in diagnosis was 28.5 days (range: 1 day-5 months). An initial misdiagnosis was documented in 5 patients (25%): conjunctivitis, *Demodex* blepharitis, warts, and pediculosis of the scalp (reported in 2 patients). Ciliary involvement by *P pubis* in children was bilateral in the majority (n = 16-3 missing data), with 1 case of monocular involvement reported. Scalp involvement by *P pubis* was found in 11 children (55%). Three children (15%) had eyebrow involvement. There were no pubertal children in this cohort and no genital involvement was reported. Symptoms of eyelid involvement by *P pubis* included ocular pruritus in 15 children (83%, 2 missing data), eyelid erythema in 7 children (44%, 4 missing data), and the presence of crusts on the free edge of the eyelid in 7 children (41%, 3 missing data) (Figure). Only one clinician had observed bluish patches on the eyelashes in a child.

**Figure:**
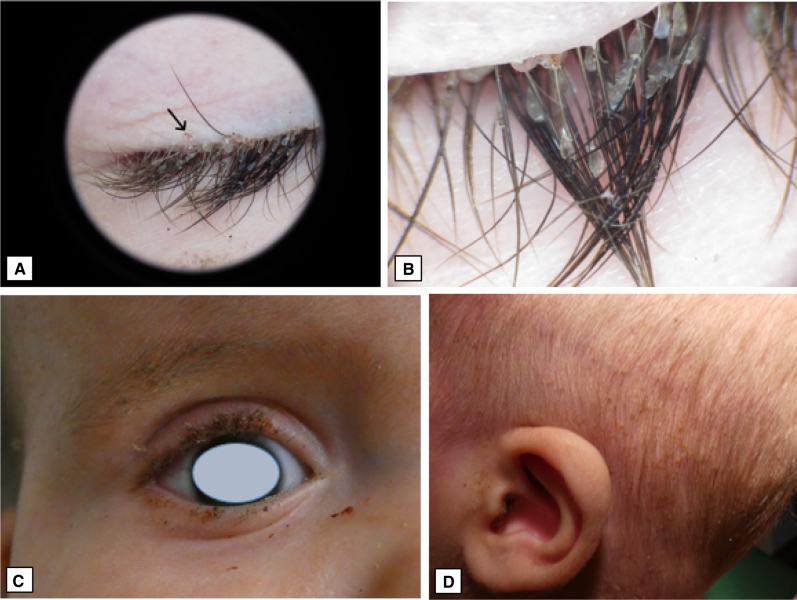
Lesions in a child with phthiriasis palpebrarum. **A,** Dermoscopic examination of eyelashes showing nits and adults of *Phthirus pubis* (*arrow*) attached to the upper eyelashes. **B,** Close-up of several nits and empty shells of *P pubis* on the eyelashes. **C,** Nits, crusts (corresponding to *P pubis* excretions), and scratching lesions. **D,** Iinfestation of the scalp by *P pubis.*

### Transmission

A case of *P pubis* infection in close contacts was identified in 12 patients (12/19 63%, 1 missing data). Among them, phthiriasis was found in the father in 8 cases (8/12; 67%) and/or in the mother in 3 cases (3/12; 25%) The existence of a chest hair infection in the father was documented on 4 cases. They were 13 children with siblings, but parasitic involvement was found in only 8 of the 13 siblings (62%). The mode of transmission has been identified in only 10 cases (53%). In 12 cases, the possibility of sexual abuse was ruled out, and in 5 cases it was not mentioned (3 missing data).

### Therapeutic Management

Two children (10%) were hospitalized for full diagnosis and treatment, respectively aged 7 months and 22 months (the youngest in the cohort). Several treatment regimens were proposed (Table). The mechanical treatment involving manual removal of nits and adult parasite was used in 16 children (80%), invariably in conjunction with other treatment modalities. The questionnaire did not specify the need for sedation for manual removal of nits and adult parasites. Chemical topical treatment was given to 15 children (75%) alone (2 patients) or in combination (13 patients). Topical treatments included vitamin A ointment, petroleum jelly, topical antibiotics (rifamycin, tobramycin), combined treatments (antibiotic [oxytetracycline] and steroid [dexamethasone]), anti-lice shampoo (malathion, permethrin), and ophthalmic irrigation solutions based on boric acid and sodium borate. Fourteen patients (70%) received oral ivermectin: 8 received a double-dose regimen (400 μg/kg), 2 received a single dose regimen (200 μg/kg), no details were provided in 4 cases. Among them, 5 children were treated with ivermectin using a 2-dose regimen with an interval of 8-10 days; information on the administration scheme is missing for the other children. One child had his hair shaved because of severe involvement. Environmental decontamination was carried out in the homes of 17 children (85%). Nineteen (95%) of the children with phthiriasis palpebrarum had a follow-up visit, of whom 16 (84%) were cured. The first 3 failed treatment lines were all different.

## Discussion

This multicenter, retrospective study with 20 patients is the largest case series of pediatric phthiriasis palpebrarum. It showed that more than one-half of patients were <5 years, no sexual abuse was evidenced, and treatments were heterogeneous. Only 4 pediatrics series are found in the literature.^11^, ^12^, ^13^, ^14^ Anane et al reported 4 children, whereas Yao reported 5 pediatric patients diagnosed with eyelash and scalp coinfestation of *P pubis*.^11^^,^^14^ Veraldi et al described 4 girls with *P pubis* infestation located exclusively on the scalp.^6^ More than one-half of the patients in the current study were subject to scalp involvement. It is possible that phthiriasis capitis is misdiagnosed as pediculosis caused by *Pediculus humanus capitis*. In our series, an initial misdiagnosis of scalp pediculosis was documented in 2 patients before the correct diagnosis.

As suggested by some authors, the identification and diagnosis of crab lice is by dermoscopy, which is a simple and noninvasive tool that can also aid in the monitoring of the treatment. In general paediatric practice, phthiriasis can usually be diagnosed with a high degree of reliability through careful clinical observation using a magnifying glass and good lighting. Examination of the lice observed on the scalp is helpful for a correct etiological diagnosis.^7^^,^^15^, ^16^, ^17^, ^18^, ^19^, ^20^, ^21^, ^22^ Phthiriasis palpebrarum may be easily misdiagnosed as blepharon-conjunctivitis as reported in the literature^23^, ^24^, ^25^, ^26^, ^27^, ^28^, ^29^, ^30^, ^31^, ^32^, ^33^, ^34^ and in our report (2 cases). The median diagnosis time was 28.5 days, with very wide extremes ranging from 1 days to 5 months. In 5 cases, the delay in diagnosis exceeded 2 months. However, the clinical presentation is relatively typical and need to be recognized. Ocular pruritus (83%), eyelid erythema (44%), and the presence of crusts that correspond to louse feces on the eyelashes (41%) are the mains characteristics of pediatric palpebral phthiriasis.^35^ Taking into account these specificities may be helpful for the early diagnosis and the identification of cases to prevent the propagation of the disease.

*P pubis* infestation is usually transmitted in adults, through close contact or sexual behavior, and occurs commonly in sexually active young people.^36^ Although nonsexual transmission is more common in children, a diagnosis of phthiriasis should always prompt a thorough evaluation to rule out the possibility of sexual abuse. Any suspicion of abuse must be reported to the relevant child protection authorities in accordance with applicable legislation.^9^^,^^10^ In the current study, the possibility of sexual abuse was ruled out in 12 cases. Although these findings may offer a degree of reassurance, this does not diminish the complexity and difficulty of diagnosing sexual abuse in children. In the current retrospective study, it is important to note that the means used to search for this mode of contamination were not specified and that the possibility of sexual abuse was even not mentioned in 5 cases. This particular mode of transmission must always be given due consideration and investigated in a systematic way. The infestation may also take place through indirect routes, such as sharing sleep arrangements with infested people or contact with fomites.^37^^,^^38^ A case of *P pubis* infection in close contacts was identified in 63% of children, mainly in fathers. Indeed, the dad's hairy torso is in contact with the eyes of small children when they are taken in their arms. It is hypothesized that patients who are hairy may act as a risk factor for the propagation of the disease. Parasitic involvement was also found in 8 of the 13 siblings (62%). The findings confirmed the need to undertake a comprehensive examination of the patients' parents and family members to ascertain the presence of *P pubis* infection.

Although the current study is the largest retrospective study on phthiriasis palpebrarum, it has some limitations. It was a retrospective study, limited by missing data and a potential recruitment bias concerning the exclusive recruitment from dermatologists, with no participation of pediatricians and ophthalmologists.

Currently, there are no standardized guidelines for treating pediatric phthiriasis palpebrarum. The variability of therapeutic approaches observed in our study reflects this lack of consensus. Recent French recommendations emphasize the use of petroleum jelly alongside mechanical removal for adults.^39^ The US Centers for Disease Control and Prevention also recommend manual removal, application of petrolatum, and educational measures to prevent reinfestation.^1^

In practice, the initial management strategy should primarily rely on local treatments for preschool and school-age children. These approaches are well tolerated and easier to implement in children. Although a significant proportion of cases in our study (80%) involved the mechanical removal of lice, this procedure can be challenging to perform in a pediatric setting because of limited cooperation and the need for precise instruments. As a result, referral to a specialist setting may be necessary.^40^, ^41^, ^42^

Systemic treatment with oral ivermectin may be considered as a second-line option, particularly in cases of treatment failure or extensive involvement.^43^, ^44^, ^45^, ^46^ Although its main advantages are ease of administration and good compliance, it is theoretically contraindicated for use in children weighing <15 kg, despite some experts using it to treat other parasitic conditions.^47^

In conclusion, ocular pruritus, eyelid erythema, and crusts on the eyelashes are highly suggestive of phthiriasis palpebrarum in the pediatric population. Searching for scalp involvement should be systematically conducted when suspecting phthiriasis palpebrarum in the pediatric population. The transmission of infestation appears to occur with a high degree of frequency through direct contact between lice from the axillary or chest hair of parents and offspring. No cases of sexual abuse have been reported in our series. This underlines that the mode of contamination of the child is, as for scabies, mainly by close family contact without forgetting that sexual abuse is still possible. To date, there is no consensus for infantile phthiriasis palpebrarum treatment, which contributes to heterogenous therapeutic strategies, and future standardization is required for optimal management.

## CRediT authorship contribution statement

**Marie Damez:** Writing – original draft, Methodology, Investigation, Formal analysis, Data curation, Conceptualization. **Anne Welfringer:** Writing – review & editing, Validation, Data curation. **Christine Leaute-Labreze:** Writing – review & editing, Validation, Data curation. **Christine Chiaverini:** Writing – review & editing, Validation, Data curation. **Stephanie Mallet:** Writing – review & editing, Validation, Data curation. **Mathilde Tardieu:** Writing – review & editing, Validation, Data curation. **Marie-Helene Jegou:** Writing – review & editing, Validation, Data curation. **Emmanuel Mahé:** Writing – review & editing, Validation, Data curation. **Annabel Maruani:** Writing – review & editing, Validation, Data curation. **Laurent Misery:** Writing – review & editing, Validation. **Claire Abasq-Thomas:** Writing – review & editing, Validation, Supervision, Methodology, Investigation, Conceptualization.

## Declaration of Competing Interest

The authors declare that they have no known competing financial interests or personal relationships that could have appeared to influence the work reported in this paper.
